# Identification of the toxic threshold of 3-hydroxybutyrate-sodium supplementation in septic mice

**DOI:** 10.1186/s40360-021-00517-7

**Published:** 2021-09-20

**Authors:** Ruben Weckx, Chloë Goossens, Sarah Derde, Lies Pauwels, Sarah Vander Perre, Greet Van den Bergh, Lies Langouche

**Affiliations:** Clinical Division and Laboratory of Intensive Care Medicine, Department of Cellular and Molecular Medicine, KU Leuven, Herestraat 49, O&N1 bus 503, 3000 Leuven, Belgium

**Keywords:** Critical illness, ICU-acquired weakness, Ketones, Toxicity, Sodium, Metabolic alkalosis

## Abstract

**Background:**

In septic mice, supplementing parenteral nutrition with 150 mg/day 3-hydroxybutyrate-sodium-salt (3HB-Na) has previously shown to prevent muscle weakness without obvious toxicity. The main objective of this study was to identify the toxic threshold of 3HB-Na supplementation in septic mice, prior to translation of this promising intervention to human use.

**Methods:**

In a centrally-catheterized, antibiotic-treated, fluid-resuscitated, parenterally fed mouse model of prolonged sepsis, we compared with placebo the effects of stepwise escalating doses starting from 150 mg/day 3HB-Na on illness severity and mortality (*n* = 103). For 5-day survivors, also the impact on *ex-vivo*-measured muscle force, blood electrolytes, and markers of vital organ inflammation/damage was documented.

**Results:**

By doubling the reference dose of 150 mg/day to 300 mg/day 3HB-Na, illness severity scores doubled (*p* = 0.004) and mortality increased from 30.4 to 87.5 % (*p* = 0.002). De-escalating this dose to 225 mg still increased mortality (*p* ≤ 0.03) and reducing the dose to 180 mg/day still increased illness severity (*p* ≤ 0.04). Doses of 180 mg/day and higher caused more pronounced metabolic alkalosis and hypernatremia (*p* ≤ 0.04) and increased markers of kidney damage (*p* ≤ 0.05). Doses of 225 mg/day 3HB-Na and higher caused dehydration of brain and lungs (*p* ≤ 0.05) and increased markers of hippocampal neuronal damage and inflammation (*p* ≤ 0.02). Among survivors, 150 mg/day and 180 mg/day increased muscle force compared with placebo (*p* ≤ 0.05) up to healthy control levels (*p* ≥ 0.3).

**Conclusions:**

This study indicates that 150 mg/day 3HB-Na supplementation prevented sepsis-induced muscle weakness in mice. However, this dose appeared maximally effective though close to the toxic threshold, possibly in part explained by excessive Na^+^ intake with 3HB-Na. Although lower doses were not tested and thus might still hold therapeutic potential, the current results point towards a low toxic threshold for the clinical use of ketone salts in human critically ill patients. Whether 3HB-esters are equally effective and less toxic should be investigated.

**Supplementary Information:**

The online version contains supplementary material available at 10.1186/s40360-021-00517-7.

## Background

Prolonged critically ill patients often suffer from ICU-acquired muscle weakness, which hampers recovery and holds risk of longer-term morbidity and mortality [1–5]. Although preventive measures such as withholding parenteral nutrition until beyond the first week in the ICU, aggressive sepsis treatment, preventing hyperglycemia and early mobilization have shown some benefit, effective therapeutic interventions are currently lacking [6–9]. In a mouse model of sepsis-induced critical illness, we have recently demonstrated that supplementation of parenteral nutrition (PN) with the ketone body 3-hydroxybutyrate (3HB) protected against loss of muscle strength [10]. In this study, a daily dose of 150 mg sodium-3HB (3HB-Na) was chosen as this is the equivalent of the amount of 3HB^−^ produced by the liver during a day of fasting [11]. 3HB-Na was administered via subcutaneous injections to avoid poor enteral absorption caused by gastrointestinal dysfunction with sepsis. In addition, this administration route avoided problems due to bad taste, nausea, abdominal cramps and diarrhea that have been reported with oral 3HB-Na intake [12]. As this first study tested only one dose of 3HB-Na, the 3HB-Na dose range effective for improving muscle force remained unknown. The toxic threshold of 3HB-Na supplementation in sepsis was not investigated, which is mandatory prior to further testing this promising intervention in human patients.

Indeed, ketone salt supplementation may hold risk, as both high amounts of sodium and of ketone acids can be toxic when clearance and/or buffer capacity is insufficient. In a non-critically ill context, excessive intake of sodium has shown to have adverse cerebrovascular consequences and to cause kidney dysfunction and fluid retention [13, 14], and hyperketonemia has been related to oxidative stress-induced liver damage, pro-inflammatory responses, cerebral and kidney abnormalities [15]. These risks could be more problematic in the context of critical illness as such patients suffer from metabolic and electrolyte disturbances, endothelial dysfunction and organ failure [16, 17]. Hence, further efficacy versus toxicity animal studies are needed to assess translational potential, if any, of 3HB-Na supplementation for the critically ill patient.

Via a dose escalation experiment - a stepwise increase or decrease of the 3HB-Na dose based on the effect on muscle force and the occurrence of adverse events – we aimed at identifying the effective dose range and the toxic/lethal dose threshold of 3HB-Na supplementation. The study was conducted in a validated and clinically relevant mouse model of prolonged sepsis-induced critical illness [18], demonstrated earlier to mimic many of the complex metabolic, endocrine and inflammatory changes of human prolonged critical illness [10, 19, 20].

## Methods

### Animal study and dosage administration

As described previously [18], we used 24-week-old C57BL/6J male mice (Janvier SAS, Chassal, France) for our validated, centrally catheterized, fluid-resuscitated model of cecal ligation and puncture. Cecal ligation and puncture is the golden standard animal model for sepsis-induced critical illness [21, 22]. A timeframe of 5 days was used as this corresponds to the prolonged phase of human critical illness [23] and is highly suitable to investigate critical illness-induced muscle weakness [10, 19].

After induction of sepsis, mice received fluid resuscitation at 0.3mL/h (colloids/crystalloids, 1:4) for the first 20 h through the central catheter. Analgesics (0.3 mg/kg buprenorphine, Vetergesic, Patheon UK Ltd, Covingham, United Kingdom) and antibiotics (16.7 mg/kg imipenem/cilastatin, Aurobindo Pharma, Saronno, VA, Italy) were administered subcutaneously twice daily throughout the 5 days study period. From day 1 onward, standard mixed parenteral nutrition at a dose of 5.8 kcal/day (Olimel N7E, Baxter, Lessines, Belgium), equivalent to 40 % of normal caloric intake, was given and supplemented twice daily with either a subcutaneous bolus injection of glucose (an isovolumetric and isocaloric dose of 187.5 mg/day, further referred to as ‘placebo’) or 3HB-Na (Sigma-Aldrich, Saint Louis, MO, USA). A dose of 150 mg/day D,L-3HB-Na was used as the reference study dose, as is the dose that was previously shown to protect against muscle weakness in septic mice [10]. Animals were housed in individual house-made transparent swivel cages and placed in a temperature-controlled (27 °C) animal cabinet with 12 h light and dark cycles. Healthy control mice were pair-fed receiving standard chow (ssniff R/M-H, ssniff Spezialdiäten Gmbh, Soest, Germany) at a daily intake comparable to the daily PN dose in septic mice (5.8 kcal/day). Mice were randomly allocated to each group. Caretakers and data collectors were blinded for group allocation. The average start bodyweight of the animals was 28.9 g ± 0.2 g SEM and not different between groups (*p* = 0.96).

**Fig. 1:**
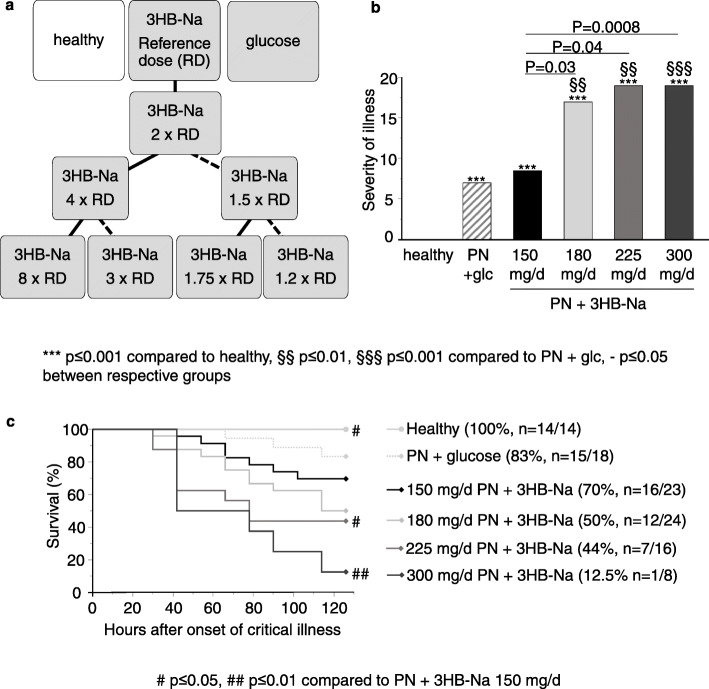
Study setup and impact of increasing doses of 3HB-Na on mortality and severity of illness. **A, **Study design. White box: healthy mice, Gray boxes: Septic mice. Full lines: increasing dose because of no increased lethality, dashed lines: decreasing dose because of increased lethality. **B, **Cumulative severity of illness of critically ill mice. Data are shown as medians. Number of animals equals total number of animals reported in panel c. glc: glucose, PN: parenteral nutrition, mg/d: mg per day. **C, **Cumulative survival of the study. Number (n) of animals is reported as n = survivors/total

Pain/discomfort was assessed twice daily by means of the Mouse Grimace Score [24], and the summed score was used as the severity of illness score. Non-survivors were allocated the maximum severity of illness score + 1. To study the dose-responses up to the toxic/lethal 3HB-Na dose threshold, we used an up-and down dosing design starting by doubling the reference dose to 300 mg/day (Fig. 1a). If toxicity was observed, the dose was systematically reduced to identify the most effective non-toxic dose that protected against muscle weakness (Fig. 1a). In the absence of toxicity (increased severity of illness or lethality), the experiment was continued until 15 animals survived up to day 5 and *ex vivo* muscle force measurements could be obtained. Exclusion criteria were physical abnormalities present before surgery (*n* = 2), pre-randomization, death during surgery (*n* = 1, from PN + 300 mg/d 3HB-Na) or catheter-malfunction during experiment (*n* = 6, 1 from PN + 300 mg/d 3HB-Na, 1 from PN + 150 mg/d 3HB-Na, 4 from PN + 225 mg/d 3HB-Na).

### Ex vivo measurement of muscle force

 After 5 days of illness, surviving mice were anaesthetized (intraperitoneal injection of 100 mg/kg ketamine, Eurovet Animal Health BV, Bladel, The Netherlands, and 13 mg/kg xylazine, V.M.D. nv/sa, Arendonk, Belgium) and euthanized via cardiac puncture and decapitation. Immediately after euthanasia, the extensor digitorum longus (EDL) muscle was isolated and placed between a fixed clamp and lever-arm (300 C-LR Dual-Mode muscle lever, Aurora Scientific, Ontario, Canada) in a temperature controlled and continuously perfused organ bath (30 °C, 95 % O_2_ − 5 % CO_2_) filled with HEPES-buffered Krebs-ringer solution (0.57 mM MgSO_4_, 10 mM glucose, 4.5 mM KCl, 120 mM NaCl, 0.7 mM Na_2_HPO_4_ dibasic, 0.9 mM Na_2_H_2_PO_4_ monobasic, 5 mM MgCl_2_, 1.2 mM KH_2_PO_4_, 2 mM CaCl_2_, 10 mM HEPES, pH 7.3). Controlled pulses of 1 A were given through two platinum electrodes to stimulate the muscle. The highest produced twitch force determined the optimal muscle length (L_0_) for each muscle separately. The maximal isometric tetanic force was measured by averaging three consecutive tetanic stimuli (180 Hz, 200 ms duration, 0.2 ms pulse width; 2 min rest intervals). The specific maximal isometric tetanic force was determined by dividing the maximal isometric tetanic force by the muscle cross-sectional area (CSA). CSA was calculated by dividing the muscle mass by the product of the density of mammalian skeletal muscle (1.06 mg/mm^3^) and the optimal fiber length (L_f_=0.44 x L_0_). Data collection was done with use of the Dynamic Muscle Analysis software (Aurora scientific).

### Blood and plasma analyses

 For surviving animals, after 5 days of illness, blood pH and blood Na^+^, K^+^, Cl^−^, HCO_3_^−^, and creatinine concentrations were measured at sacrifice with use of the Epoc® Blood Analysis System (Siemens Healthineers, The Hague, The Netherlands). Whole blood 3HB^−^ concentrations were measured with the StatStrip Xpress®2 Glucose/Ketone meter (Nova Biomedical, Waltham, MA, USA) 30 min after injection of the study dose on day 1. In plasma collected at sacrifice, 3HB^−^ was quantified with a commercial enzymatic kit (EnzyChrom™ ketone body assay kit, Bioassay Systems, Hayward, CA, USA). Impact of 3HB-Na on inflammation was assessed by quantification of plasma TNFα (Mouse TNF-alpha Quantikine HS ELISA Kit, R&D systems, Minneapolis, MN, USA). As ketone bodies can suppress lipolysis which may have detrimental effects during sepsis [10, 19], plasma free fatty acids (Free Fatty Acid Fluorometric Assay, Cayman, Ann Arbor, MI, USA) and glycerol (Glycerol Assay Kit, Sigma-Aldrich) were quantified. Plasma aldosterone (All species Aldosterone ELISA Kit, LSBio, Seattle, WA, USA) was quantified as a marker of the renin-angiotensin-aldosterone system, which is involved in fluid retention and can be affected by salt intake.

### Tissue analyses

 For surviving animals, after 5 days of illness, water content of liver, brain, lung and muscle biopsies was determined by a freeze-drying process. Tissue samples were weighed, dried at 95 °C for 6 h and weighed again. To determine gene expression of markers of tissues damage and inflammation, RNA was extracted from liver and kidney samples using the RNeasy mini RNA isolation kit (Qiagen, Hilden, Germany) and from hippocampi with the NucleoSpin RNA mini kit (Macherey-Nagel, Dueren, Germany). Liver and kidney were homogenized in Qiazol (Qiagen) and hippocampi in mercaptoethanol-supplemented RP1 buffer (Macherey-Nagel) at 6.500 rpm for 45 s with ceramic beads in a Precellys 24 machine (Bertin Technologies, Villeurbanne, France), followed by use of respective kits according to the manufacturer’s instructions. RNA concentrations were quantified by Nanodrop spectrophotometer (ND-1000, Nanodrop Technologies, Wilmington, DE, USA) and reverse-transcribed using Superscript III Reverse Transcriptase (Invitrogen, Merelbeke, Belgium) and random primers (Invitrogen). Real-time quantification of cDNA was performed with StepOne Plus (Applied Biosystems, Carlsbad, CA, USA) using commercial TaqMan assays (Applied Biosystems) for all gene expression analyses (supplementary Table 1). Data are shown normalized to hypoxanthine-guanine phosphoribosyltranferase (*Hprt*) and were expressed as a fold change of the mean of control mice.

### Histological analyses

For animals surviving the 5 days of sepsis, hematoxylin and eosin stained formalin fixed paraffin tissue sections were used to semi-quantitatively assess changes in histological structure. Liver sections were scored for feathery appearance of cytoplasm, loss of structure, sinusoidal dilatation and infiltration of inflammatory cells [25]. To assess brain damage, hippocampal regions CA1, CA3 and dentate gyrus were scored for presence of damaged neurons, identified as neurons with shrunken eosinophilic cytoplasm and pyknotic nuclei [26]. The hippocampus was evaluated as it is the brain region most susceptible to damage during experimental sepsis and it is vulnerable to stress-induced damage [27–30]. Tissue sections were scored as 0, 1 or 2 for minimal (< 10 %), mild (10–20 %) or severe (> 20 %) abundance of aforementioned parameters. Scoring was performed by two independent observers who reached consensus in case of scoring discrepancies. Hippocampal microglia were stained with a rabbit anti-Iba1 polyclonal antibody (1:500, No.019-19741, Fuijifilm Wako chemicals, Richmond, VA, USA), followed by HRP-linked polyclonal goat anti-rabbit antibody (1:100, No.P0448, Dako, Glostrup, Denmark) and visualization with DAB (Dako). The number of microglia per mm^2^ were counted in the CA1, CA3 and dentate gyrus regions with ImageJ software.

### Statistical analyses

 Statistical analyses were performed with use of JMP Pro 14 (SAS Institute, Cary, USA). Data are presented as box plots with interquartile ranges and whiskers describing the 25th and 75th percentiles or as bars with standard error of the means. To compare differences between study groups, Student’s t-test, Wilcoxon, Log-Rank and Pearson’s chi-squared test were used, as appropriate. Two-sided p-values of ≤ 0.05 were considered significant.

## Results

### Effect of increasing 3HB-Na doses on severity of illness scores and mortality

Severity of illness scores and mortality of septic mice receiving the reference dose of 150 mg 3HB-Na per day were comparable to those of septic mice receiving placebo (Fig. 1b, c). Doubling of the daily dose to 300 mg 3HB-Na increased severity of illness scores 2.2-fold (Fig. 1b) and mortality increased from 30.4 to 87.5 % (Fig. 1c). Given these toxic/lethal effects, the daily dose was subsequently reduced to 1.5 times the reference dose (225 mg/day 3HB-Na). As compared with the reference 150 mg/day, also this dose increased severity of illness scores and mortality (Fig. 1b, c). A further reduction of the dose to 1.2 times the reference dose (180 mg/day 3HB-Na) also doubled severity of illness scores as compared with the reference 150 mg/day 3HB-Na dose, while mortality was no longer significantly different (*p* = 0.2 vs. 150 mg/day 3HB-Na) (Fig. 1b,c). As only 7/16 mice receiving 225 mg/day 3HB-Na and only 1/8 mice receiving 300 mg/day 3HB-Na survived until the preplanned end of the experiment (day 5), these mice were combined in one group for further plasma and tissue analyses.

### Effect of increasing doses of 3HB-Na on blood electrolytes, acid-base balance and tissue water content

Bolus injections of increasing doses of 3HB-Na resulted in progressively higher plasma concentrations of 3HB^−^, as measured 30 min after injection, from mean ± SEM 3.45 ± 0.27 mM 3HB^−^ at 150 mg/day, 4.46 ± 0.26 mM at 180 mg/day and 4.88 ± 0.37 mM at 225 mg/day. However, 3HB^−^ appeared to be rapidly cleared from the circulation as 4 h post injection on day 5, plasma 3HB^−^ concentrations were no longer higher than in the placebo group in any of the supplemented groups (supplemented: mean ± SEM 0.17 ± 0.04 mM 3HB^−^ vs. placebo: 0.19 ± 0.04 mM 3HB^−^; *p* ≥ 0.8).

**Fig. 2:**
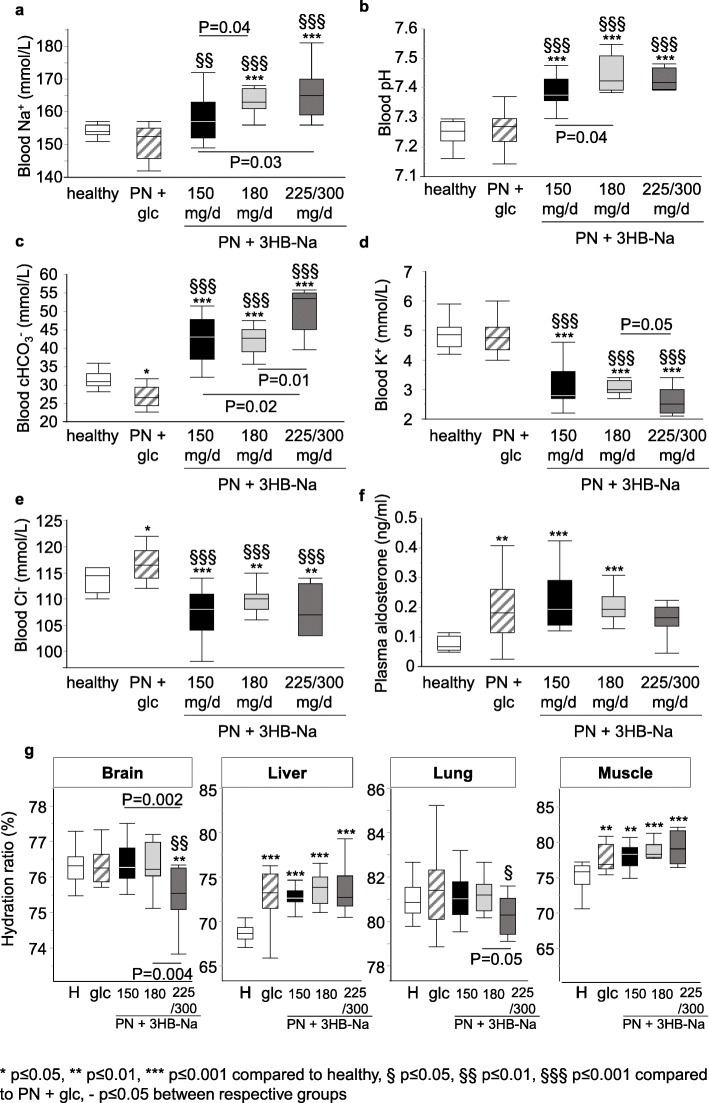
Impact of increasing doses of 3HB-Na on blood ion balance, aldosterone and tissue water content. Whole blood taken at sacrifice was assessed for **A**, Blood Na^+^; **B**, Blood pH; **C**, Blood calculated HCO_3_^−^; **D**, Blood K^+^; **E**, Blood Cl^−^; and **F**, plasma aldosterone; **G**, Water content was determined in brain, liver, lung and muscle. Data are shown as median and interquartile range. Number (n) of animals: white, healthy (*n* = 14); dashes, PN + glucose (*n* = 15); PN + 3HB-Na: black, 150 mg/d (*n* = 16); gray, 180 mg/d (*n* = 12); dark gray, 225/300 mg/d (*n* = 8). H: healthy, glc: glucose, PN: parenteral nutrition, mg/d: mg per day

Given its formulation as a salt, increasing 3HB-Na doses may affect electrolytes and acid-base balance through the accompanying increase in Na^+^-load. Indeed, supplementation of 150, 180, 225 and 300 mg 3HB-Na per day corresponded with a respective daily Na^+^-intake of 37.05, 45.76, 52.06 and 66.75 mg, as compared with 12.96 mg per day intake in the placebo group. Blood Na^+^-concentrations were dose-dependently increased in all septic mice receiving 3HB-Na (Fig. 2a). In addition, all groups of septic mice receiving 3HB-Na revealed a dose-dependent rise in blood pH as compared with healthy control mice and as compared with placebo-treated septic mice (Fig. 2b). Blood HCO_3_^−^ was increased in all 3HB-Na-treated septic mice compared to those treated with placebo and healthy control mice (Fig. 2c). A dose of 225/300 mg/day 3HB-Na further increased blood HCO_3_^−^ as compared to the other 3HB-Na-treated septic groups (Fig. 2c). Blood Cl^−^ and K^+^-concentrations were lowered in all 3HB-Na treated septic groups as compared with healthy control mice and as compared with placebo-treated septic mice (Fig. 2d,e). The decrease in Cl^−^ occurred independently of the dose of 3HB-Na, whereas blood K^+^ was lowest with the 2 highest 3HB-Na doses (225/300 mg/day).

To rule out the involvement of the renin-angiotensin-aldosterone system in the clearly disturbed blood electrolytes with increasing 3HB-Na doses, plasma aldosterone was quantified. However, plasma aldosterone was equally elevated in septic groups as compared with healthy control mice and independently of the dose of 3HB-Na (Fig. 2f).

As hyperketonemia has been linked to cerebral edema and sodium intake can weigh heavily on the fluid balance by inducing water retention, we next measured the water content of vital organs and tissues. Both liver and muscle of septic mice contained more water than of healthy control mice, but this water content was unaffected by any of the 3HB-Na doses (Fig. 2g). In contrast, water content of brain and lung was lowered in septic mice supplemented with the highest doses of 225/300 mg/day 3HB-Na (Fig. 2g).

### Effect of increasing doses of 3HB-Na on markers of tissue damage in brain, liver and kidney

**Fig. 3:**
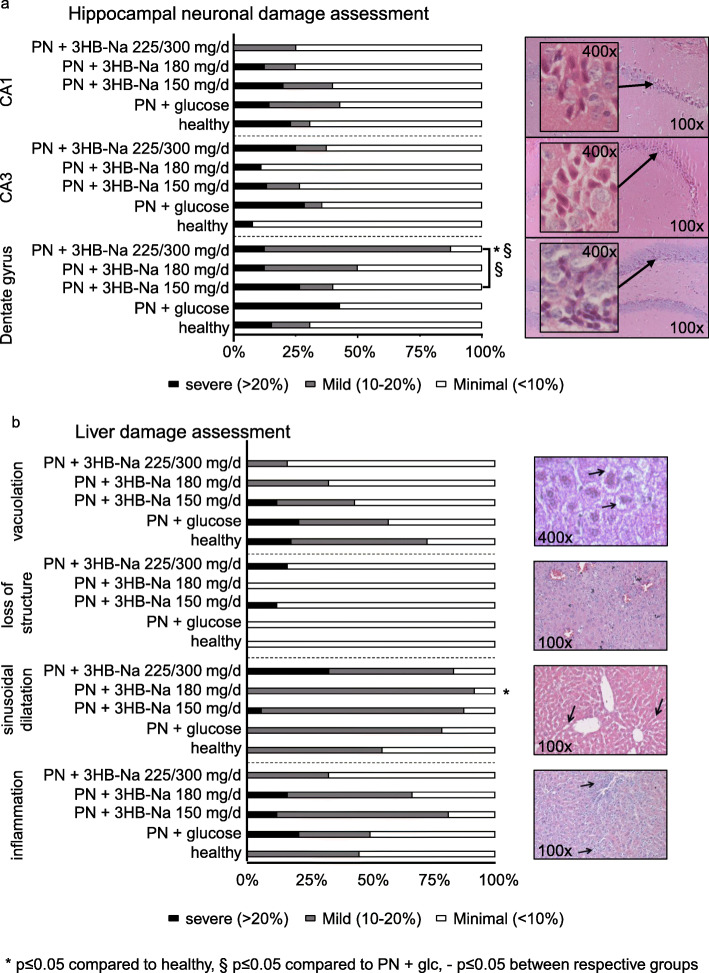
Semi-quantitative histological assessment of **a** hippocampal neuronal damage and **b** liver damage. Data are shown as cumulative percentages of the respective group. Number (n) of animals: healthy (*n* = 13); PN + glucose (*n* = 14); PN + 3HB-Na: 150 mg/d (*n* = 16); 180 mg/d (*n* = 12); 225/300 mg/d (*n* = 8). PN: parenteral nutrition, glc: glucose, mg/d: mg per day

As brain, liver and kidney are susceptible to damage induced by high sodium concentrations and by hyperketonemia, markers of tissue damage were assessed in these vital organs. No histological signs of neuronal damage were observed in hippocampal CA1 and CA3 regions during sepsis, nor with increasing 3HB-Na doses (Fig. 3a). In contrast, neuronal damage in the dentate gyrus became apparent with increasing 3HB-Na doses (Fig. 3a). Liver histology showed no significant differences in vacuolation, loss of structure and inflammation among all groups of septic mice and healthy controls (Fig. 3b). Hepatic sinusoidal dilatation was also generally unaffected by sepsis, with only an increase observed in septic mice receiving 180 mg 3HB-Na per day as compared with healthy control mice (Fig. 3b).

**Fig. 4:**
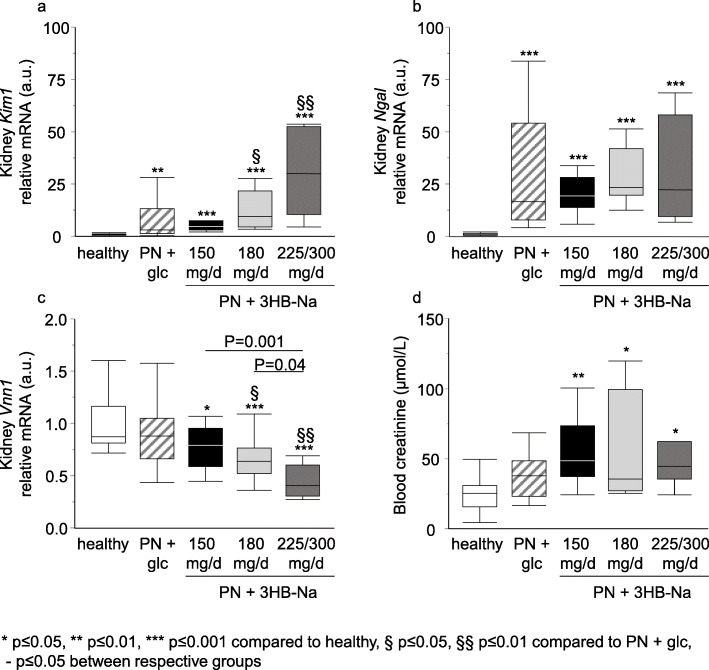
Impact of increasing doses of 3HB-Na on markers associated with kidney damage**. a-c** Relative gene expression of **a**, *Kim1*; **b**, *Ngal*; **c**, *Vnn1*; and **d**, blood creatinine levels at sacrifice. Number (n) of animals: white, healthy (*n* = 14); dashes, PN + glucose (*n* = 15); PN + 3HB-Na: black, 150 mg/d (*n* = 16); gray, 180 mg/d (*n* = 12); dark gray, 225/300 mg/d (*n* = 8). glc: glucose, PN: parenteral nutrition, mg/d: mg per day

As markers of kidney damage, relative gene expression of *Kim1*, *Ngal* and *Vnn1* were measured. Both *Kim1* and *Ngal* mRNA levels were higher in septic mice than in healthy control mice (Fig. 4a, b). 3HB-Na injection resulted in a dose-dependent increase in *Kim1* and dose-dependent decrease in *Vnn1*, whereas *Ngal* expression was not affected by the 3HB-Na dose (Fig. 4a-c). Similar to *Kim1* and *Ngal*, blood creatinine levels were also higher in all 3HB-Na treated septic mice than in healthy control mice, whereas placebo-treated septic mice were no different from controls (Fig. 4d).

### Effect of increasing doses of 3HB-Na on ketone-regulated pathways

**Fig. 5:**
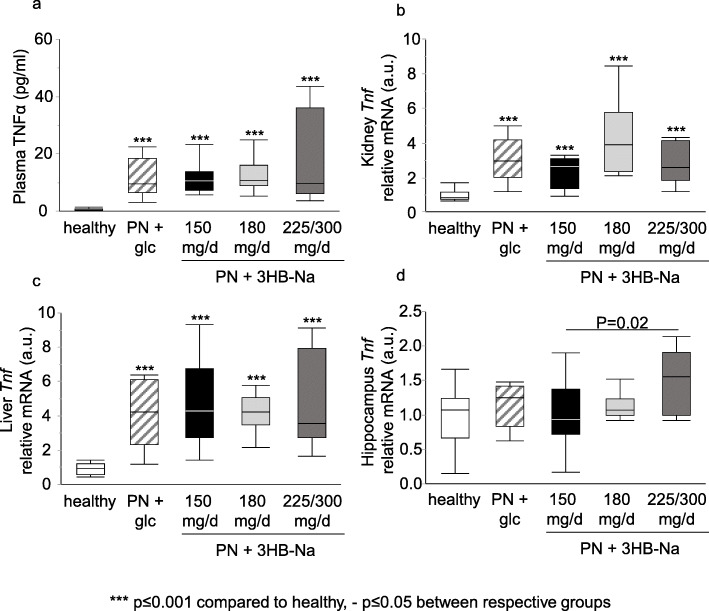
Impact of increasing doses of 3HB-Na on markers of inflammation.** a** Plasma levels of TNFα. Relative mRNA expression of *Tnf* measured in **b** kidney, **c** liver and **d** hippocampus. **a**-**d** Number (n) of animals: white, healthy (*n* = 14); dashes, PN + glucose (*n* = 15); PN + 3HB-Na: black, 150 mg/d (*n* = 16); gray, 180 mg/d (*n* = 12); dark gray, 225/300 mg/d (*n* = 8). glc: glucose, PN: parenteral nutrition, mg/d: mg per day

As 3HB^−^ has known anti-inflammatory properties, possibly counteracted by excess sodium intake, we investigated circulating and tissue markers of inflammation. Plasma TNFα concentration and *Tnf* gene expression in kidney and liver were higher in all septic groups than in healthy control mice, but unaffected by 3HB-Na supplementation (Fig. 5a-c). In the hippocampus, *Tnf* gene expression was only increased in septic mice supplemented with the highest doses (225 and 300 mg/day) 3HB-Na as compared with the reference dose of 150 mg/day 3HB-Na (Fig. 5d). In addition, in septic mice treated with 180 mg/day 3HB-Na and with 225/300 mg/day 3HB-Na, the number of microglia was increased in the hippocampal CA3 and dentate gyrus regions as compared with healthy control mice and as compared with placebo-treated septic mice (Supplementary Fig. 1).

**Fig. 6:**
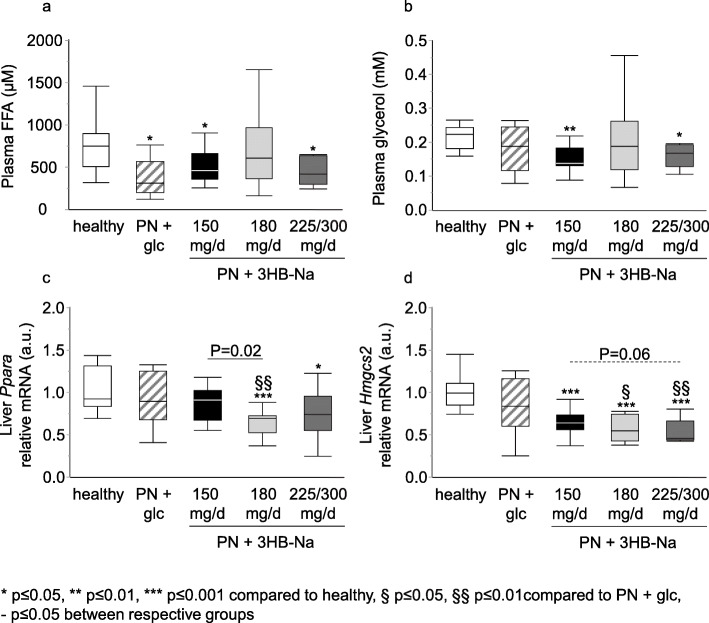
Dose-dependent effect of 3HB-Na supplementation on markers linked to ketone metabolism**.** Plasma levels of **a** free fatty acids and **b** glycerol. Liver relative mRNA expression of **c** *Ppara* and **d** *Hmgcs2*. **a-d** Number (n) of animals: white, healthy (*n* = 14); dashes, PN + glucose (*n* = 15); PN + 3HB-Na: black, 150 mg/d (*n* = 16); gray, 180 mg/d (*n* = 12); dark gray, 225/300 mg/d (*n* = 8). glc: glucose, PN: parenteral nutrition, mg/d: mg per day

Ketone bodies can suppress lipolysis which may have detrimental effects during sepsis [10]. Plasma free fatty acid and glycerol concentrations were reduced with sepsis, however largely unaffected by 3HB-Na supplementation (Fig. 6a, b). In contrast to circulating markers of lipolysis, hepatic mRNA levels of key ketogenic enzymes *Ppara* and *Hmgcs2* were dose-dependently decreased in 3HB-Na supplemented mice as compared with healthy control mice (Fig. 6c, d).

### Effect of increasing doses of 3HB-Na on muscle weakness and muscle wasting

**Fig. 7:**
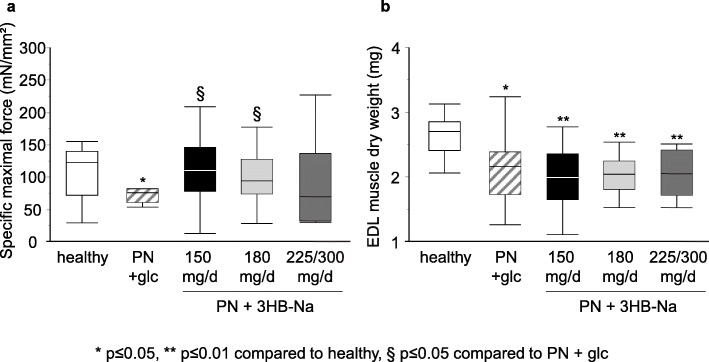
Impact of increasing doses of 3HB-Na on muscle force and wasting in septic mice.** A**, *Ex vivo* EDL muscle force measurements presented as specific force (peak tetanic tension per unit muscle mass). White, healthy (*n* = 13); dashes, PN + glc (*n* = 12); PN + 3HB-Na: black, 150 mg/d (*n* = 13); light gray, 180 mg/d (*n* = 12); gray, 225/300 mg/d (*n* = 8). **B**, EDL muscle dry weight. White, healthy (*n* = 13); dashes, PN + glc (*n* = 15); PN + 3HB-Na: black, 150 mg/d (*n* = 16); light gray, 180 mg/d (*n* = 12); gray, 225/300 mg/d (*n* = 8). **A, B** Data are shown as median and interquartile range. glc: glucose, PN: parenteral nutrition, mg/d: mg per day

As was also observed previously [10], the placebo-treated septic mice suffered from a substantial loss of specific maximal muscle force as compared with healthy control mice. Specific maximal muscle force of septic mice treated with 150 mg/day 3HB-Na was increased as compared with placebo-treated septic mice, and this up to the level of the healthy control mice (Fig. 7a). Also 180 mg/day 3HB-Na increased muscle force as compared with placebo-septic mice, an effect that was similar to that of the reference dose of 150 mg/day 3HB-Na (Fig. 7a). Among the 8 surviving septic mice treated with 225/300 mg/day 3HB-Na, the measured specific muscle force was highly variable and did not differ from that of healthy control mice nor from other septic groups (Fig. 7a). EDL muscle mass was lower in all septic mice than in healthy control mice, irrespective of 3HB-Na supplementation (Fig. 7b).

## Discussion

In a mouse model of sepsis-induced critical illness, we investigated toxicity versus efficacy to prevent muscle weakness of increasing doses of 3HB-Na supplementation. A daily dose of 150 mg 3HB-Na was previously shown to safely prevent muscle weakness without increased risk of morbidity or mortality, a finding that was confirmed here. Raising this reference dose stepwise, up to a doubling, revealed toxicity as evidenced by a dose-dependent aggravated hypernatremia, hypokalemia and metabolic alkalosis, by kidney damage, dehydration of brain and lungs, hippocampal neuronal damage and inflammation, and by increased mortality. A maximally protective effect on muscle force was observed with the reference 3HB-Na dose of 150 mg/day, a dose that appeared very close to the toxic threshold.

While treating septic mice with a dose of 150 mg of 3HB-Na per day was previously shown to prevent muscle weakness without increasing risk of morbidity or mortality [10], as was confirmed here, it was striking to find that increasing the daily dose of 3HB-Na by 20 % caused increased illness severity and further increasing the dose by 50–100 % caused increased mortality. Biochemically, as compared with 150 mg/day of 3HB-Na, 180 mg/day of 3HB-Na or more evoked more pronounced metabolic alkalosis and more severe hypernatremia. Metabolism of 3HB-Na involves its separation into 3HB^−^ and Na^+^. The observed metabolic alkalosis could be explained by intracellular uptake and metabolism of 3HB^−^ while the accompanying Na^+^ was retained in the circulation. Indeed, we found that 3HB^−^ was rapidly cleared from the circulation. Intracellular uptake of 3HB^−^ requires cotransport of protons as co-factors via the monocarboxylate transporters [31]. In addition, the accumulation of the positive strong Na^+^ ions in the circulation while the negative 3HB^−^ ions were removed increased the strong ion difference in the blood, which may directly contribute to, or be a major cause of metabolic alkalosis [32, 33]. In turn, metabolic alkalosis can cause hypoxia, hypoperfusion and arrhythmia, which may have contributed to the poor outcome with use of the higher 3HB-Na doses.

Alternatively, hypokalemia, hypochloremia and increased aldosterone levels are in itself potential contributors to metabolic alkalosis [34]. Although hypokalemia and hypochloremia were observed with 3HB-Na treatment of the septic mice, these changes were more likely a consequence than a cause of the alkalosis. Metabolic alkalosis can indeed cause extra-renal shift in potassium from the extracellular to the intracellular fluid compartment and chloride ions can be passively exchanged along with hydrogen ions in order to maintain electroneutrality [35, 36]. However, hypokalemia and hypochloremia were also present in the septic group treated with 150 mg/day of 3HB-Na, where no increase in morbidity and mortality was observed. Theoretically, high sodium intake inhibits systemic RAAS by reducing the renin secretion in the juxtaglomerular apparatus, which would result in decreased aldosterone levels [37]. While all septic mice expectedly displayed sepsis-induced high plasma aldosterone concentrations [38], 3HB-Na intake did not further affect aldosterone concentrations, arguing against a role of the RAAS system in the observed changes in potassium and chloride levels.

In higher doses, 3HB-Na caused kidney damage, as evidenced by a rise in plasma creatinine and a dose-dependent response in the kidney damage markers *Kim1* and *Vnn1*. This kidney damage was probably not a consequence of hyper-inflammation, as 3HB-Na doses did not affect plasma TNFα nor renal *Tnf* expression. Both high sodium intake and excess ketone acids could directly cause kidney dysfunction [15, 39, 40]. However, toxicity studies performed with 3HB-esters, which increase ketone bioavailability without necessitating co-administration of a Na^+^ cation, argue against 3HB^−^ being the cause of the kidney damage [41, 42]. In the studies of 3HB-esters performed in rats, no toxic side effects were observed up to doses of 15 g/kg bodyweight [41]. Also in studies of human volunteers, 3HB-ester-induced high plasma 3HB^−^ concentrations, reaching levels of 3.30 mM, were found to be safe [42]. Furthermore, in diabetic mice, exogenously elevating circulating 3HB^−^ to approximately 1.5 mM has shown to protect against kidney failure [43]. Hence, the adverse effect on the kidney documented in the septic mice by administering 180–300 mg/day 3HB-Na (6–10 g/kg bodyweight/day) point to Na^+^ overload rather than hyperketonemia as the causal factor. The dehydration of brain and lungs with higher 3HB-Na doses is in line with a deleterious impact of the high sodium load, as excessively high ketone levels are more likely to evoke cerebral edema [13, 15].

Besides the kidney, also liver and brain were assessed for signs of damage, since excessive intake of sodium and/or hyperketonemia have been demonstrated to target these tissues [13–15]. Where no additional damage was observed with 3HB-Na supplementation in liver tissue, the hippocampal brain region displayed increased inflammatory markers and damaged neurons with the highest doses of 3HB-Na. In rodents and humans, both a high sodium intake [44] and hyperketonemia [15] have been linked to brain damage and cognitive impairment. However, it should be noted that hyperketonemia-associated brain damage was only observed in the presence of diabetic ketoacidosis with severe hyperglycemia [15]. Our septic mice did not present with acidosis nor with hyperglycemia. The ability of 3HB-esters to improve cognitive performance in mice (receiving 500 mg/day) and in human patients suffering from Alzheimer’s disease [42, 45, 46] further argues against adverse effects by hyperketonemia, and support the possibility of sodium-overload induced fluid shifts as a culprit.

Adverse effects of increasing doses of 3HB-Na also did not appear to be related to the known suppressive effects of ketones on lipolysis [47, 48]. The already low plasma FFA and glycerol in septic mice were not further suppressed by 3HB-Na supplementation, whereas a dose-dependent decrease in *Ppara* and *Hmgcs2* expression occurred, two key components of ketogenesis. These data point to a normal physiological negative feedback control with exogenous 3HB without causing detrimentally low lipid levels [10, 19, 49].

Importantly, we here confirmed that a daily dose of 150 mg 3HB-Na was effective in protecting against sepsis-induced muscle weakness in mice [10]. Higher 3HB-Na doses did not further amplify the effect on muscle force, which suggest that a maximal effect was reached. As we did not test doses lower than 150 mg 3HB-Na per day, we cannot exclude that a lower dose might have maintained efficacy while further reducing the risk on toxic side effects.

## Conclusions

3HB-Na supplementation attenuated sepsis-induced muscle weakness in mice with a maximal protective effect at a daily dose of 150 mg. However, this dose was close to toxic threshold. Toxicity may have been at least partially explained by the excess sodium load with increasing 3HB-Na doses. These findings confirm the potential of 3HB as a strategy to prevent ICU-acquired weakness, but strongly argue against the use of a salt-form. Whether pharmacological 3HB formulations that do not increase sodium load, such as 3HB-esters, are equally effective and less toxic remains to be investigated.

## Supplementary Information

**Additional file 1 **: **Supplementary Table 1.** Genes used.

**Additional file 2 **: **Supplementary Fig. 1**. Hippocampal microglia count. Quantitative measurement of microglia in the dentate gyrus, CA1 and CA3 of the hippocampus. White, healthy (*n*=14); dashes, PN + glucose (*n*=15); PN + 3HB-Na: black, 150 mg/d (*n*=16); gray, 180 mg/d (*n*=10); dark gray, 225/300 mg/d (*n*=8). glc: glucose, PN: parenteral nutrition, mg/d: mg per day.

## Data Availability

The datasets used during the current study are available from the corresponding author on reasonable request.

## Abbreviations

3HB: 3-hydroxybutyrate
3HB-Na: 3-hydroxybutyrate-sodium-salt
CSA: Cross-sectional area
EDL: Extensor digitorum longus
FFA: Free fatty acids
HPRT: Hypoxanthine-guanine phosphoribosyltransferase
ICU: Intensive care unit
ICUAW: Intensive care unit acquired weakness
L0: Optimal muscle length
Lf: Optimal fiber length
PN: Parenteral nutrition
