# Supplementary material for: Identification of the toxic threshold of 3-hydroxybutyrate-sodium supplementation in septic mice
Source: BMC Pharmacol Toxicol. 2021 Sep 20;22:50. doi: 10.1186/s40360-021-00517-7 (PMC8454128; doi:10.1186/s40360-021-00517-7)
Supplement: Supplementary file 1 — Additional file 1 : Supplementary Table 1. Genes used. [file 40360_2021_517_MOESM1_ESM.docx]

**Supplementary table 1. Genes used**

| **Gene symbol** | **Alternative name** | **Product name**  **(Applied biosystems)** |
| --- | --- | --- |
| Hprt | hypoxanthine guanine phosphoribosyl transferase | Mm00446968_m1 |
| Tnf | Tumor necrosis factor alpha | Mm00443258_m1 |
| Havcr1 | Kidney injury molecule 1 (Kim1) | Mm00506686_m1 |
| Lcn2 | Neutrophil gelatinase-associated lipocalin (Ngal) | Mm01324470_m1 |
| Vnn1 | Vanin 1 | Mm00495970_m1 |
| Ppara | Peroxisome proliferator activated receptor alpha | Mm00440939_m1 |
| Hmgcs2 | 3-hydroxyl-3-methylglutaryl-Coenzyme A synthase 2 | Mm00550050_m1 |
