# Supplementary figures and images for: Identification of the toxic threshold of 3-hydroxybutyrate-sodium supplementation in septic mice
Source: BMC Pharmacol Toxicol. 2021 Sep 20;22:50. doi: 10.1186/s40360-021-00517-7 (PMC8454128; doi:10.1186/s40360-021-00517-7)

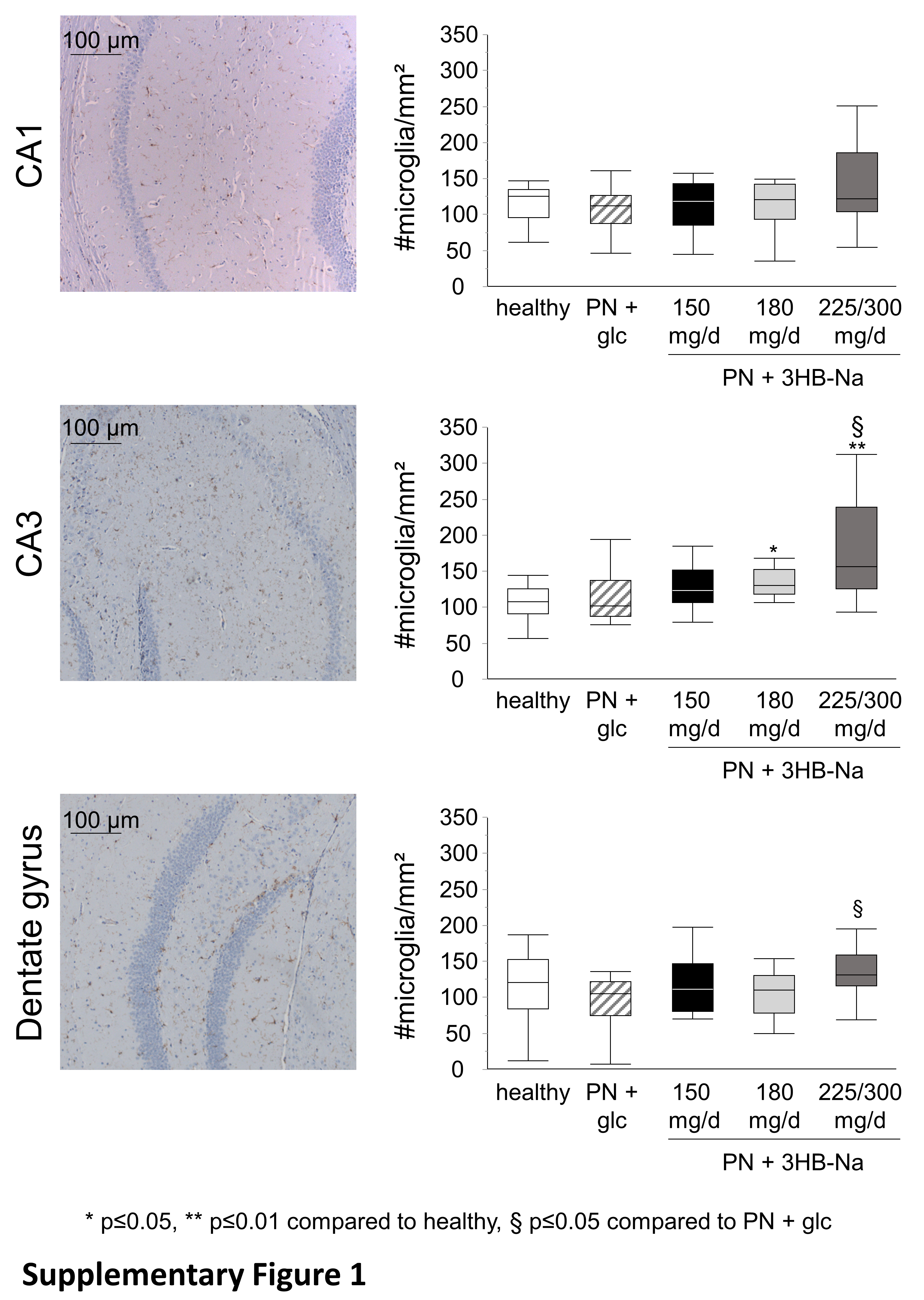

Supplement: Supplementary file 2 — Additional file 2 : Supplementary Fig. 1. Hippocampal microglia count. Quantitative measurement of microglia in the dentate gyrus, CA1 and CA3 of the hippocampus. White, healthy (n=14); dashes, PN + glucose (n=15); PN + 3HB-Na: black, 150 mg/d (n=16); gray, 180 mg/d (n=10); dark gray, 225/300 mg/d (n=8). glc: glucose, PN: parenteral nutrition, mg/d: mg per day. [file 40360_2021_517_MOESM2_ESM.tif]
